# TREM2‐IGF1 Mediated Glucometabolic Enhancement Underlies Microglial Neuroprotective Properties During Ischemic Stroke

**DOI:** 10.1002/advs.202305614

**Published:** 2023-12-27

**Authors:** Sheng Yang, Chuan Qin, Man Chen, Yun‐Hui Chu, Yue Tang, Luo‐Qi Zhou, Hang Zhang, Ming‐Hao Dong, Xiao‐Wei Pang, Lian Chen, Long‐Jun Wu, Dai‐Shi Tian, Wei Wang

**Affiliations:** ^1^ Department of Neurology Tongji Hospital Tongji Medical College Huazhong University of Science and Technology Wuhan 430030 China; ^2^ Hubei Key Laboratory of Neural Injury and Functional Reconstruction Huazhong University of Science and Technology Wuhan 430030 China; ^3^ Department of Neurology Mayo Clinic Rochester MN 55905 USA

**Keywords:** ischemic stroke, microglia, oxidative phosphorylation, Trem2‐Igf1 signaling pathway

## Abstract

Microglia, the major resident immune cells in the central nervous system, serve as the frontline soldiers against cerebral ischemic injuries, possibly along with metabolic alterations. However, signaling pathways involved in the regulation of microglial immunometabolism in ischemic stroke remain to be further elucidated. In this study, using single‐nuclei RNA sequencing, a microglial subcluster up‐regulated in ischemic brain tissues is identified, with high expression of Igf1 and Trem2, neuroprotective transcriptional signature and enhanced oxidative phosphorylation. Microglial depletion by PLX3397 exacerbates ischemic brain damage, which is reversed by repopulating the microglia with high Igf1 and Trem2 phenotype. Mechanistically, Igf1 serves as one of the major down‐stream molecules of Trem2, and Trem2‐Igf1 signaling axis regulates microglial functional and metabolic profiles, exerting neuroprotective effects on ischemic stroke. Overexpression of Igf1 and supplementation of cyclocreatine restore microglial glucometabolic levels and cellular functions even in the absence of Trem2. These findings suggest that Trem2‐Igf1 signaling axis reprograms microglial immunometabolic profiles and shifts microglia toward a neuroprotective phenotype, which has promising therapeutic potential in treating ischemic stroke.

## Graphical abstract

Trem2‐Igf1 signaling axis plays pivotal roles in microglial activities against ischemic stroke injuries. Upon ischemic stroke, microglia up‐regulate Trem2 expression levels, which subsequently boosts Igf1 expression and affects microglial metabolic profiles as well as cellular functions. Regulation of Igf1 can eventually exert effects on microglial metabolism, especially oxidative phosphorylation, which consequently affects microglial activities and alleviates ischemic stroke injuries.

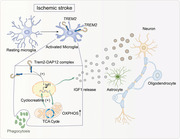

## 1 Introduction

Ischemic stroke is majorly characterized as an immediate blockade of cerebral blood flow, which subsequently causes lack of oxygen and glucose in the brain and induces injuries in various cells in the central nervous system (CNS).^[^ ^1^ ^]^ The mechanisms of ischemic stroke potentially involve a plethora of pathophysiology processes and related signaling pathways, many of which are associated with metabolic reprogramming and neuroinflammation processes. Importantly, microglia, as the major resident immune cells in the CNS, are reportedly involved in the whole pathogenesis of ischemic stroke, especially neuroinflammation, along with a variety of phenotypic and functional alterations.^[^ ^2^ ^]^ Recent studies with growing evidence showed that microglia, other than simply mediating neuroinflammation and propagation of hypoxia‐reperfusion injuries, are also key players in both structural and functional recovery after ischemic stroke.^[^ ^2^, ^3^ ^]^ Therefore, it's of great significance to describe the heterogeneity of microglial activation and their distinct functions in the progress, especially identifying the protective subset and underlying mechanism.

Microglia actively participate in an ever‐growing list of biological processes, including brain development, immune defense, and ceaseless monitoring of the brain parenchyma for dysfunction, infection, or damage.^[^ ^4^ ^]^ To ensure constant participation during ischemic stroke, microglia rapidly extend and retract their processes, migrate to the lesion site, engulf and degrade cellular debris, and release various cytokines and chemokines.^[^ ^2^ ^]^ These motility and activities are likely to require high energy demand that is essential for their functions.^[^ ^4^ ^]^ Although some literature covers the link between metabolic reprogramming and immune function in peripheral macrophages and CNS microglia,^[^ ^4^, ^5^ ^]^ much less is currently known about how to modulate microglial phenotype toward neuroprotective ones via regulation of energy metabolism in ischemic stroke.

To draw a link between microglial diverse responses and metabolic alterations in ischemic stroke, as well as to investigate the underlying mechanisms, we performed a series of experiments both in vivo and in vitro. Using single‐nuclei RNA sequencing (snRNA‐seq), we initially identified a subcluster of microglia upregulated in experimental ischemic stroke mice, with a characteristic profile of significantly elevated levels of Igf1 and Trem2, enhanced oxidative phosphorylation (OXPHOS) and cellular phagocytosis activities. Furthermore, Trem2 is proved to be involved in these microglial activation processes, in which Igf1 may serve as an important downstream molecule, contributing to neuroprotective effects through interactions with neighboring cells such as neurons, astrocytes and oligodendrocytes. The Trem2‐Igf1 signaling pathway underpins microglial metabolism under cerebral ischemia‐reperfusion circumstances, in which overexpression of Igf1 alone or supplementation of cyclocreatine could enhance microglial oxidative phosphorylation levels, and consequently promote microglial proliferation and exert neuroprotective properties, even in the absence of Trem2.

## 2 Results

### 2.1 A Microglial Subcluster Identified in Mouse Brain after MCAO

**Figure 1:**
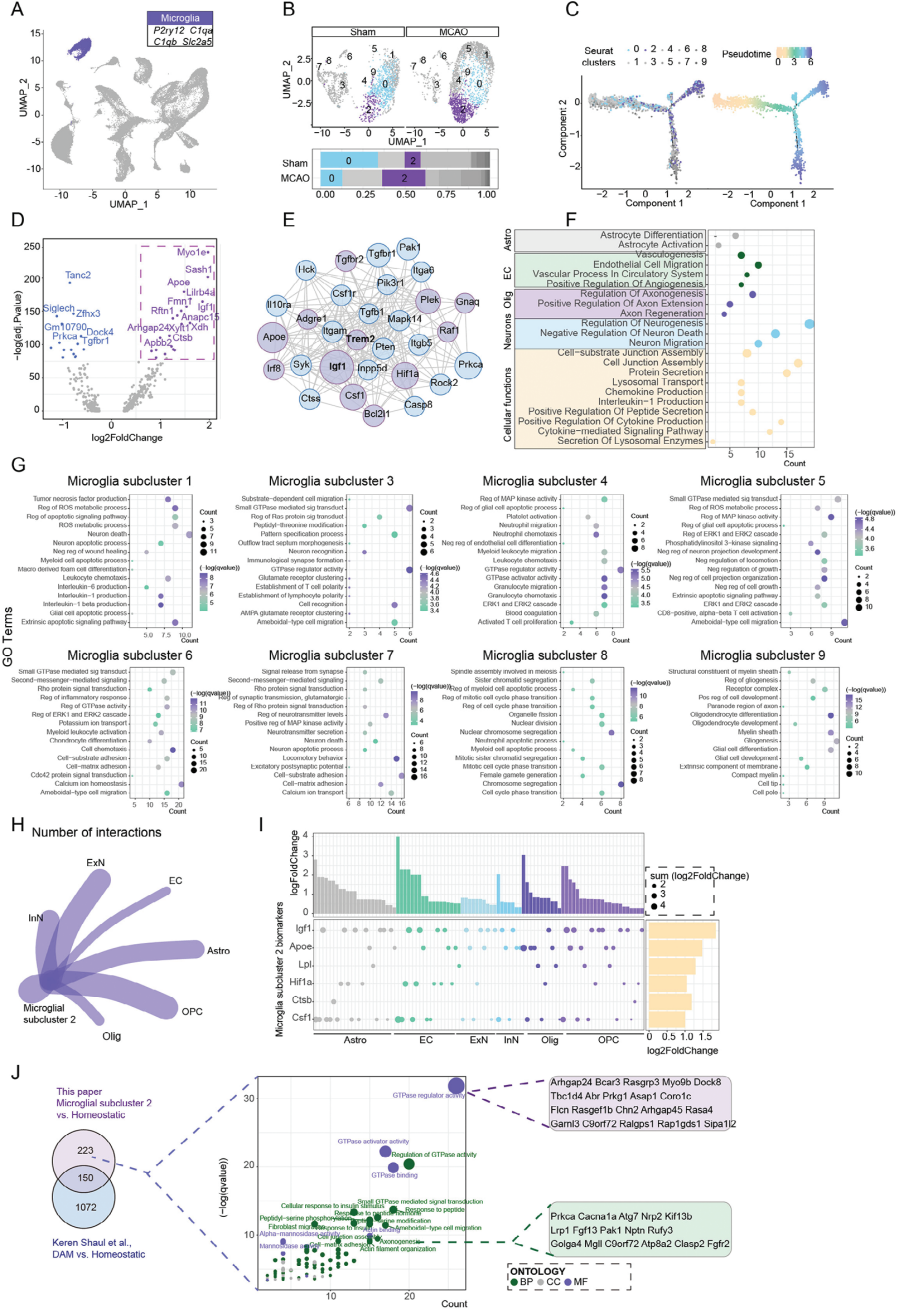
A microglial subcluster, identified in ischemic hemisphere after MCAO in mice. A) UMAP and unsupervised clustering of all cells and identification of microglia using specific microglial biomarkers. B) Re‐clustering of microglia, with subcluster 2 and subcluster 0 specifically colorized. Proportions of different microglial subclusters between Sham group and MCAO group. C) Pseudotime analysis of microglia clusters. Subcluster 2 and subcluster 0 were highlighted with different colors. D,E) Volcano plots and PPI analysis depicting differentially expressed genes (DEGs) between microglial subcluster 2 and subcluster 0. F) GO biological processes of the DEGs with respect to activities of other cell types and microglial functions under the threshold of p value < 0.05 and q value < 0.05. G) GO terms enriched for different microglial subclusters under the threshold of p value < 0.05 and q value < 0.05. H) CellChat interactions of microglia subcluster 2 with other cells. I) STRING analysis revealing ligand‐receptor interactions between microglial subcluster 2 and other cells. J) Comparison of the transcriptional profiles of microglial subcluster 2 and disease‐associated microglia (DAM).

To characterize the heterogeneity of microglial activation after ischemic stroke, snRNA‐Seq was performed on cells extracted from mouse ischemic brain after MCAO post day 7 and sham group. In total, we obtained single‐nuclei transcriptomes of 30 828 cells from 3 MCAO mice, and 30 598 cells from 3 sham‐operated mice. After basic annotation, dimensionality reduction of snRNA‐seq data by Uniform Manifold Approximation and Projection (UMAP) identified 24 cell clusters, including 2 microglia clusters, 1 oligodendrocyte cluster, 1 oligodendrocyte precursor cell cluster, 1 astrocyte cluster, 1 endothelial cell cluster, 7 inhibitory neuron clusters and 11 excitatory neuron clusters (**Figure** **1**A; Figure S1A,B, Supporting Information). Microglia were identified with canonical microglial markers including P2ry12, C1qb, C1qa and Slc2a5 (Figure 1A; Figure S1A, Supporting Information).^[^ ^6^ ^]^ Fine‐resolution re‐clustering of microglia showed these cells could be further divided into 10 subclusters (Figure 1B). By comparing the microglial subclusters in MCAO group and sham group, we found that the proportion of microglial subcluster 2 was significantly increased after MCAO, while that of subcluster 0 was greatly downregulated (Figure 1B). Meanwhile, pseudotime analysis showed that the subcluster 2 and 0 were distributed at two distinctive positions of pseudotime trajectory (Figure 1C). In parallel, volcano plot showed that subcluster 2 microglia expressed those elevated genes related to phagocytosis and activation, such as Igf1, Ctsb, Trem2 and Apoe, while subcluster 0 microglia were more likely to express homeostatic genes, such as Tgfbr1, Siglech and P2ry12 (Figure 1D; Figure S1C,D, Supporting Information). Among the differentially expressed genes between the two subclusters, we identified Trem2 and Igf1 to be the pivotal hub genes via PPI analysis (Figure 1E).

Gene ontology (GO) enrichment of biological processes further indicated that microglial subcluster 2 was closely associated with various key neuroprotective functions, including migration, differentiation, genesis, and activation toward other types of cells, suggesting its potential role in interacting with other cell types and contributing to CNS tissue repair. Additionally, microglial subcluster 2 is also associated with the secretion of neurotrophic factors, chemokines, cytokines, and tight junction assembly (Figure 1F). These results suggest that microglial subcluster 2 potentially affects CNS microenvironment via secretion of various factors and promotion of tissue regeneration, thereby contributing to CNS repair after ischemic stroke. Furthermore, we conducted a comprehensive analysis of the potential roles played by other microglial subclusters. Microglial subcluster 1 is majorly associated with the release of pro‐inflammatory cytokines, signals related to ROS, and apoptotic processes in neurons and glial cells. Subclusters 3, 4, 5 and 6 are mostly involved in GTPase activity and other kinase cascade signals, such as the MAPK signaling pathways, with the participation of immune cell chemotaxis and glial cell apoptotic process. Subcluster 7 is mainly linked to synaptic transmission processes and neuronal death, while subcluster 8 is involved in cellular division and cell cycle. Subcluster 9 is involved in glial cell genesis, development and differentiation (Figure 1G).

In line with these results, CellChat cell interaction and STRING analysis revealed that the microglial subcluster 2 possibly communicated with astrocytes, endothelial cells, neurons, oligodendrocytes and OPCs through ligand‐receptor interactions, mainly relying on Igf1, Apoe, Lpl, Hif1a, Ctsb and Csf1 (Figure 1H,I). We also identified the existence of microglial subcluster with similar transcriptional signatures, including elevated expressions of Trem2 and Igf1 in two recent scRNA‐seq datasets from MCAO mice at 1 day and 3 days post MCAO.^[^ ^7^ ^]^ Likewise, these microglial subclusters with similar transcriptional signatures in these datasets exhibited potentially neuroprotective properties toward the surrounding cells, providing evidence of the robustness and reproducibility of our findings (Figure S2, Supporting Information). Besides, we have also compared the transcriptional signature of microglial subcluster 2 with DAM,^[^ ^8^ ^]^ WAM^[^ ^9^ ^]^ and PAM.^[^ ^10^ ^]^ The results revealed that microglial subcluster 2 in sn‐RNA‐Seq shares both common and exclusive genes with the other microglial subclusters, and these exclusively expressed genes are closely related with cellular repair processes (Figure 1J; Figure S3A,B, Supporting Information). Conclusively, the transcriptional profiles of microglia suggested increased frequency of a subcluster during ischemic stroke, with molecular signature of upregulated Trem2 and Igf1, and functional features of neuroprotective and pro‐repairing properties.

### 2.2 Increased Expression of Igf1 and Trem2 in Microglia with Enhanced Glucometabolic Levels and Cellular Activity

**Figure 2:**
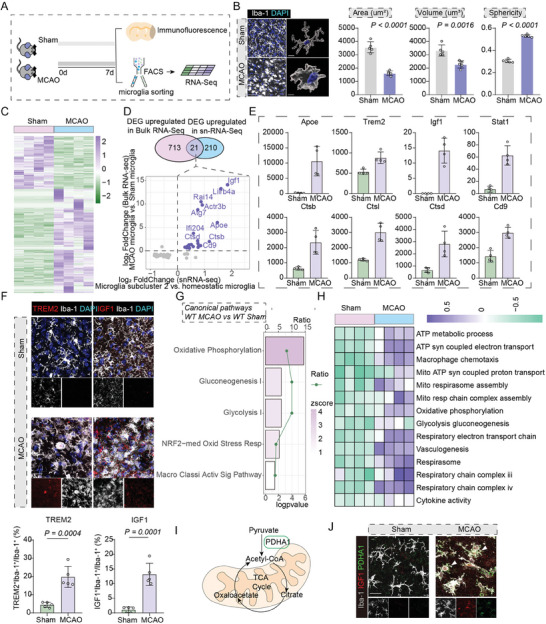
Activated microglial phenotype with enhanced glucometabolic levels and cellular activity. A) Schematic image of the workflow. B) Immunofluorescence staining and morphological analysis of Iba‐1^+^ microglia from both Sham‐operated group and MCAO ischemic lesions. Scale Bar: 50 µm for the left immunofluorescence plots, 10 µm for reconstructed 3D plots. N = 5 per group. Data are expressed as mean ± SD, Unpaired t‐test. C) Heatmap of all the differentially expressed genes between microglia from MCAO group and Sham‐operated group under the threshold of qvalue < 0.05 and absolute value of log2FoldChange > 0.5. D) Venn diagrams indicate significantly differentially expressed genes in each comparison, and report overlapping genes in Bulk‐RNA‐Seq and sn‐RNA‐Seq. E) Barplots showing the indicated gene expression in microglia sorted from Sham group and MCAO mice. F) Immunofluorescence staining and proportions of TREM2^+^ Iba‐1^+^ and IGF1^+^ Iba‐1^+^ cells in microglia. Scale Bar: 20 µm. G) IPA analysis of the DEGs identified between microglia sorted from wild‐type MCAO and sham‐operated group. H) Gene set variation analysis of the DEGs identified between microglia sorted from wild‐type MCAO and sham‐operated group. I) A brief plot depicting tricarboxylic acid cycle. Key enzyme such as Pyruvate Dehydration A (PDHA1) is highlighted. J) Representative images of immunofluorescence staining of PDHA1^+^IGF1^+^Iba‐1^+^ in ischemic lesions and Sham brains. Scale Bar: 20 µm.

To characterize the features of microglia in ischemic brain, we depicted the dynamic changes in microglial morphology and density after MCAO. By immunofluorescence, we found that microglia increased remarkably and accumulated surrounding the infarct lesions, characterized by smaller cellular area/volume and higher sphericity (Figure 2B; Figure S4, Supporting Information). Concurrently, cellular sphericity and proportion of TREM2^+^ microglia peaked at 7 days post ischemia (**Figure** **2**A,B; Figure S4, Supporting Information).^[^ ^11^ ^]^ We then sorted microglia by FACS at day 7 post MCAO and performed bulk RNA‐Seq to further elucidate the transcriptional profiles of microglia in ischemic stroke. In total, there were 21 upregulated genes both in microglial subcluster 2 in sn‐RNA‐Seq (versus homeostatic microglia) and microglia from MCAO mice (versus microglia from sham mice), including Igf1, Trem2, Apoe and several phagocytosis‐related genes, such as Ctsb and Ctsl (Figure 2C‐E). Additionally, immunofluorescence staining revealed the significantly elevated proportion of IGF1^+^ and TREM2^+^ microglia after ischemia (Figure 2F; Figure S4B–E, Supporting Information), further confirmed those molecular signatures identified by snRNA‐seq and Bulk‐RNA‐Seq analysis.

Furthermore, the results from both Gene set variation analysis (GSVA) and Ingenuity pathway analysis (IPA) revealed that, compared to the Sham‐operated group, the transcriptional profiles of microglia from MCAO ischemic hemispheres exhibited the upregulated levels of oxidative phosphorylation, ATP metabolic processes, glycolysis, as well as microglia or macrophage activation or chemotaxis activities, which was in line with previous studies^[^ ^12^ ^]^ (Figure 2G,H). Moreover, immunofluorescence results revealed that the expression levels of pyruvate dehydrogenase alpha 1 (PDHA1), a key enzyme in the tricarboxylic acid cycle (TCA cycle) in microglia, was significantly upregulated after ischemia, which co‐localized with up‐regulated IGF1 in microglia, showing their specific expression in the aforementioned microglial subcluster (Figure 2I,J).

### 2.3 Igf1 and Trem2 High‐Expressing Microglia Play Important Roles in Neurological Recovery Following Ischemic Stroke

**Figure 3:**
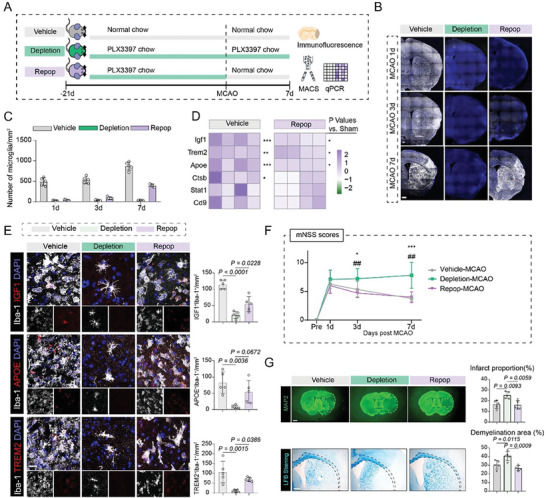
Microglia expressing Trem2 and Igf1 were indispensable in neurological recovery following ischemic stroke. A) Schematic plot illustrating three strategies adopted in this part of experiment. B,C) Immunofluorescence staining and quantitative analysis of Iba‐1^+^ microglia density in the ischemic hemisphere of Vehicle, depletion and repopulation mice at different time points (1 day, 3 days and 7 days) after surgery. N = 5 per group. Scale Bar: 500 µm. D) Heatmap showing microglial subcluster 2 genes expression detected by RT‐PCR. Vehicle versus Sham and Repop versus Sham. * P < 0.05, ** P < 0.01, *** P < 0.001, N = 4 per group. one‐way ANOVA followed by Bonferroni's post hoc test. E) Immunofluorescence staining and density of IGF1^+^ Iba‐1^+^, APOE^+^ Iba‐1^+^ and TREM2^+^ Iba‐1^+^ cells in microglia in Vehicle group, Depletion group and Repopulation group. Scale Bar: 20 µm. N = 5 per group. Data are expressed as mean ± SD, one‐way ANOVA followed by Bonferroni's post hoc test. F) Neurological deficits were evaluated using mNSS score. N = 10‐12 per group. Data are expressed as mean ± SD, one‐way ANOVA followed by Bonferroni's post hoc test, * P < 0.05 Vehicle versus Depletion group, ***P < 0.001 Vehicle versus Depletion group, ## P < 0.01 Repop versus Depletion group. G) Immunofluorescence staining of MAP2 and LFB staining of three different groups in the ischemic hemisphere of Vehicle, depletion and repopulation mice at 7 days post MCAO. Scale Bar: 1 mm. N = 5 per group. Data are expressed as mean ± SD, one‐way ANOVA followed by Bonferroni's post hoc test.

To investigate functional relevance of Igf1 and Trem2 positive microglial subcluster in disease progression, microglia ablation approach was used in experimental ischemic stroke. To this end, three different strategies were adopted (**Figure** **3**A): 1) Mice were given normal chow pre‐and post‐MCAO (Vehicle group); 2) Mice were constantly given PLX3397‐formulated chow for microglia elimination (Depletion group); and 3) Mice were given PLX3397‐formulated chow for 21 days before MCAO, then switched to normal chow for microglial repopulation (Repop group). Oral PLX3397 administration led to gross microglial depletion (>90% reduction; Figure 3B,C). However, microglial density in Repop group progressively increased and reached ≈60% of the Vehicle group at day 7 post MCAO (Figure 3B,C). Then microglia in different groups were sorted to detect the expression levels of indicated genes. Interestingly, we found that repopulated microglia exhibited upregulated gene expression of Trem2, Igf1, Apoe compared to microglia from sham mice (Figure 3D). Additionally, results from immunofluorescence analysis indicated a significant and substantial decrease in the density of TREM2^+^, IGF1^+^ and APOE^+^ microglia in the PLX3397 group compared to the vehicle chow group, with ≈90% of the population depleted. However, in the microglia repopulation group, the density of microglia positive for TREM2 and IGF1 was largely recovered (Figure 3E).

In addition, we found that mice with sustained microglial depletion exhibited more extensive loss of MAP2 staining, LFB staining, and higher mNSS score, indicating exacerbated infarction, demyelination, and more severe neurological deficits (Figure 3F,G), aligned with a previous study.^[^ ^13^ ^]^ Interestingly, when microglia repopulated, these detrimental effects were largely reversed. Conclusively, these data suggested that depletion of microglia exacerbates ischemic stroke‐induced injuries, while repopulation of microglia exerts neuroprotective effects. The strategy of microglial depletion and repopulation likely acts through depletion and repopulation of IGF1 and TREM2 positive microglial subpopulation, which serves as a potential therapeutic approach in ischemic stroke.

### 2.4 Trem2‐Igf1 Axis Underlies Microglial Immunometabolism and Neuroprotective Function in Ischemic Stroke

**Figure 4:**
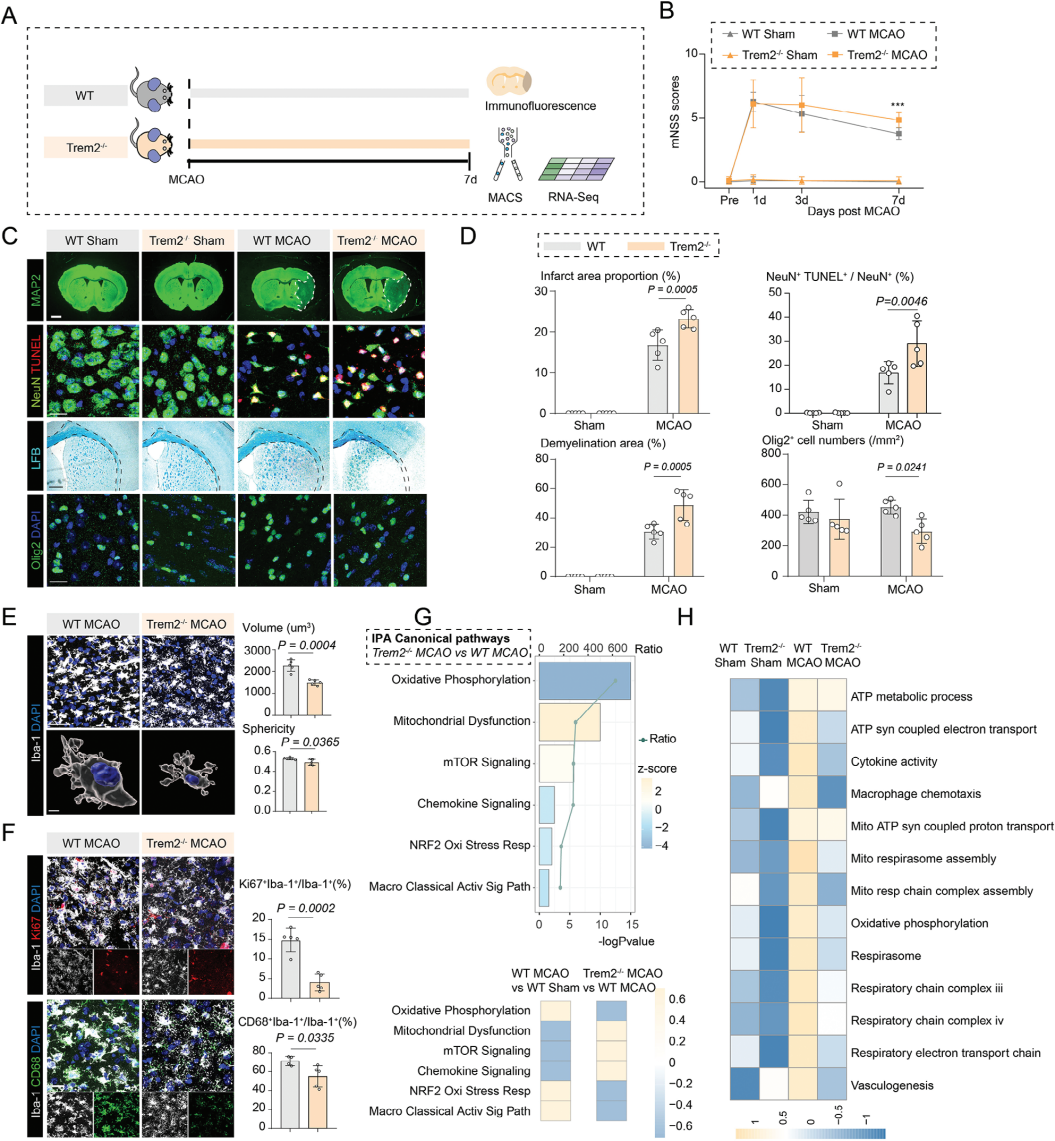
Trem2 deficiency exacerbates ischemic injury and dampens microglial functions. A) Schematic plot illustrating the workflow of this part of experiment. B) Neurological deficits were evaluated using mNSS score. N = 12 per group. Data are expressed as mean ± SD, two‐way ANOVA followed by Bonferroni's post hoc test, ***P < 0.001 versus the WT MCAO group. C) Immunofluorescence staining of MAP2, TUNEL^+^ NeuN^+^ apoptotic neurons, Olig2^+^ oligodendrocytes and LFB staining in mice of wild‐type sham, Trem2^–/–^ sham, wild‐type MCAO, and Trem2^–/–^ MCAO groups. Scale Bar: 1 mm for MAP2 staining and LFB staining, and 20 µm for the rest. D) Quantitative analysis of infarct area proportion, TUNEL^+^ NeuN^+^ apoptotic neurons proportion, demyelination area proportion and Olig2^+^ oligodendrocytes density. N = 5 per group. Data are expressed as mean ± SD, two‐way ANOVA followed by Bonferroni's post hoc test. E) Immunofluorescence staining and morphological analysis of Iba‐1^+^ microglia in wild‐type MCAO, and Trem2^−/−^ MCAO mice. Scale Bar: 20 µm for immunofluorescence and 5 µm for 3D reconstruction. N = 5 per group. Data are expressed as mean ± SD, unpaired t‐test. F) Immunofluorescence staining and quantitative analysis of Iba‐1^+^Ki67^+^ proliferative microglia and Iba‐1^+^CD68^+^ phagocytic microglia in ischemic lesions of wild‐type and Trem2^−/−^ mice. Scale Bar: 20 µm. N = 4–5 per group. Data are expressed as mean ± SD, unpaired t‐test. G) Ingenuity pathway analysis (IPA) of the differentially expressed genes between Trem2^−/−^ MCAO and WT MCAO microglia. Pathways up‐regulated in Trem2^−/−^ MCAO were marked in yellow and pathways down‐regulated were marked in blue. H) Gene set variation analysis (GSVA) of biological processes and pathways related with microglial activities and aerobic respiration in microglia from wild‐type sham, Trem2^−/−^ sham, wild‐type MCAO, and Trem2^−/−^ MCAO groups.

In line with our RNA‐seq analysis, immunostaining results further confirmed that TREM2^+^ microglia increased largely after MCAO and mainly accumulated in the peri‐infarct areas (Figure S4C‐E, Supporting Information). Notably, we demonstrated more apoptotic neurons, less Olig2^+^ cells, larger ischemic lesions/demyelinated area, and more severe neurological deficits were found in Trem2^–/–^ MCAO mice compared with the wild‐type MCAO mice (**Figure** **4**A‐D). Accordingly, microglia in Trem2 deficiency also exhibited smaller cellular volume and lower sphericity (Figure 4E), defective properties of proliferation (Iba‐1^+^ Ki67^+^) and phagocytosis (Iba‐1^+^CD68^+^) (Figure 4F). Ingenuity pathway analysis (IPA) of differentially expressed genes between Trem2^–/–^ and WT MCAO microglia revealed that, knockout of Trem2 contributes to downregulation of oxidative phosphorylation and microglial activation processes, while it concurrently exacerbates mitochondrial dysfunction (Figure 4G). GSVA of the four groups also shows that the levels of aerobic respiration and microglial activity associated pathways are mostly up‐regulated in microglia from Wild‐type MCAO mice compared with those from wild‐type controls, while these pathways have been dampened after Trem2 knockout (Figure 4H). Collectively, our results indicated that Trem2 deficiency could exacerbate ischemic brain damage, potentially via dampening microglial activities.

**Figure 5:**
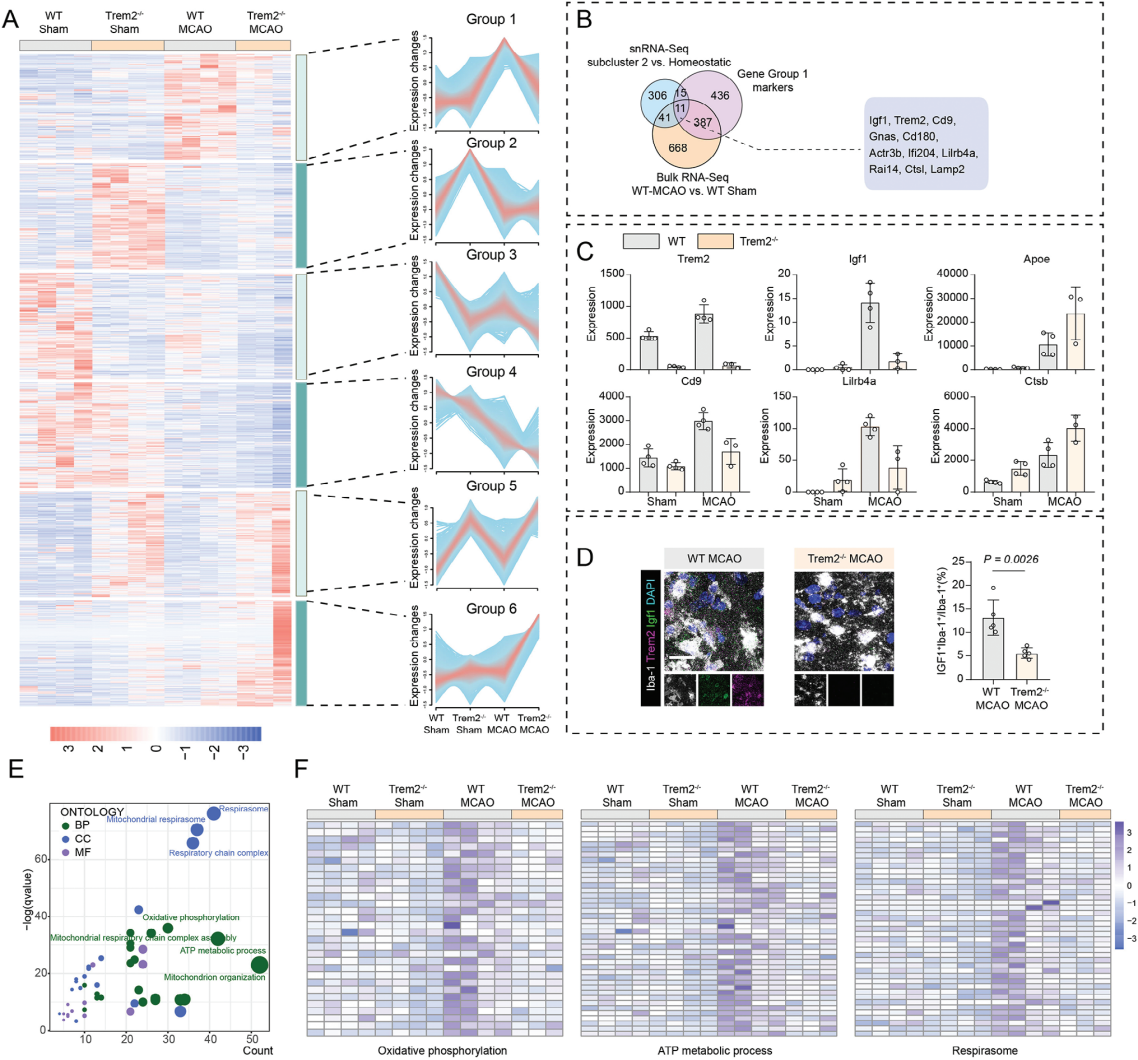
Trem2‐Igf1 signaling axis possibly regulates microglial metabolism and functions in ischemic stroke. A) Trend analysis of the Bulk‐RNA‐Seq data of the sorted microglia from the four groups (Wild‐type sham, Trem2^−/−^ sham, Wild‐type MCAO, Trem2^−/−^ MCAO). B) Venn diagrams indicate significantly differentially expressed genes in each comparison, and report 11 overlapping genes in Bulk‐RNA‐Seq, sn‐RNA‐Seq and group 1 identified in trend analysis. C) Barplots showing the indicated gene expression in microglia sorted from wild‐type sham, Trem2^−/−^ sham, wild‐type MCAO, and Trem2^−/−^ MCAO mice. Data are expressed as mean ± SD, N = 3–4 per group. D) Immunofluorescence staining of TREM2^+^ IGF1^+^ Iba‐1^+^ microglia in wild‐type MCAO and Trem2^−/−^ MCAO mice. Scale Bar: 20 µm. N = 5 per group. Data are expressed as mean ± SD, unpaired t‐test. E) Gene Ontology enrichment analysis of biological processes based on the genes identified between microglia sorted from wild‐type MCAO and Trem2^−/−^ MCAO mice. Biological processes with p value < 0.05 and q value < 0.05 are considered statistically significant. F) Heatmap depicting expression levels of genes related with oxidative phosphorylation, ATP metabolic pathways and Respirasome in microglia sorted from four groups (Wild‐type sham, Trem2^−/−^ sham, Wild‐type MCAO, Trem2^−/−^ MCAO).

To further illustrate the downstream signals of Trem2 in microglia after ischemic stroke, we conducted a trend analysis in the bulk RNA‐Seq data. The differentially expressed genes were then clustered and grouped based on expression patterns (**Figure** **5**A). Group 1 represented those genes that were upregulated in wild‐type mice after MCAO but remained nearly unchanged in Trem2^–/–^ group, i.e, dependent of Trem2 (Figure 5A). By comparing the Group 1 genes and the potential markers for microglial subcluster 2 in sn‐RNA‐Seq, an overlap of 11 genes, including Trem2, Igf1 and Ctsl, were identified (Figure 5B). Among them, the change in Igf1 expression was most notable, increased by over 2^10‐fold after MCAO, but largely diminished in Trem2 deficiency (Figure 5C). Consistently, immunofluorescence results revealed that IGF1^+^ microglia mainly accumulated within ischemic boundary zone in WT MCAO group, but relatively lower in Trem2^–/–^ MCAO mice (Figure 5D). In addition, group 1 genes also showed several enriched pathways related to cellular aerobic respiration, ATP production and metabolism, which were downregulated in Trem2 deficient microglia (Figure 5E,F). Taken together, Trem2 functions as a pivotal regulator of microglial activities and aerobic respiration in ischemic stroke, which affects microglial morphology, phenotypes and functions. Deficiency in Trem2 may dampen microglial aerobic respiration processes and physiological functions, which subsequently exacerbates ischemic stroke‐induced brain injuries.

### 2.5 Overexpression of Igf1 or Supplementation of Cyclocreatine Boosts Microglial OXPHOS and Shows Neuroprotective Effects in the Absence of Trem2

**Figure 6:**
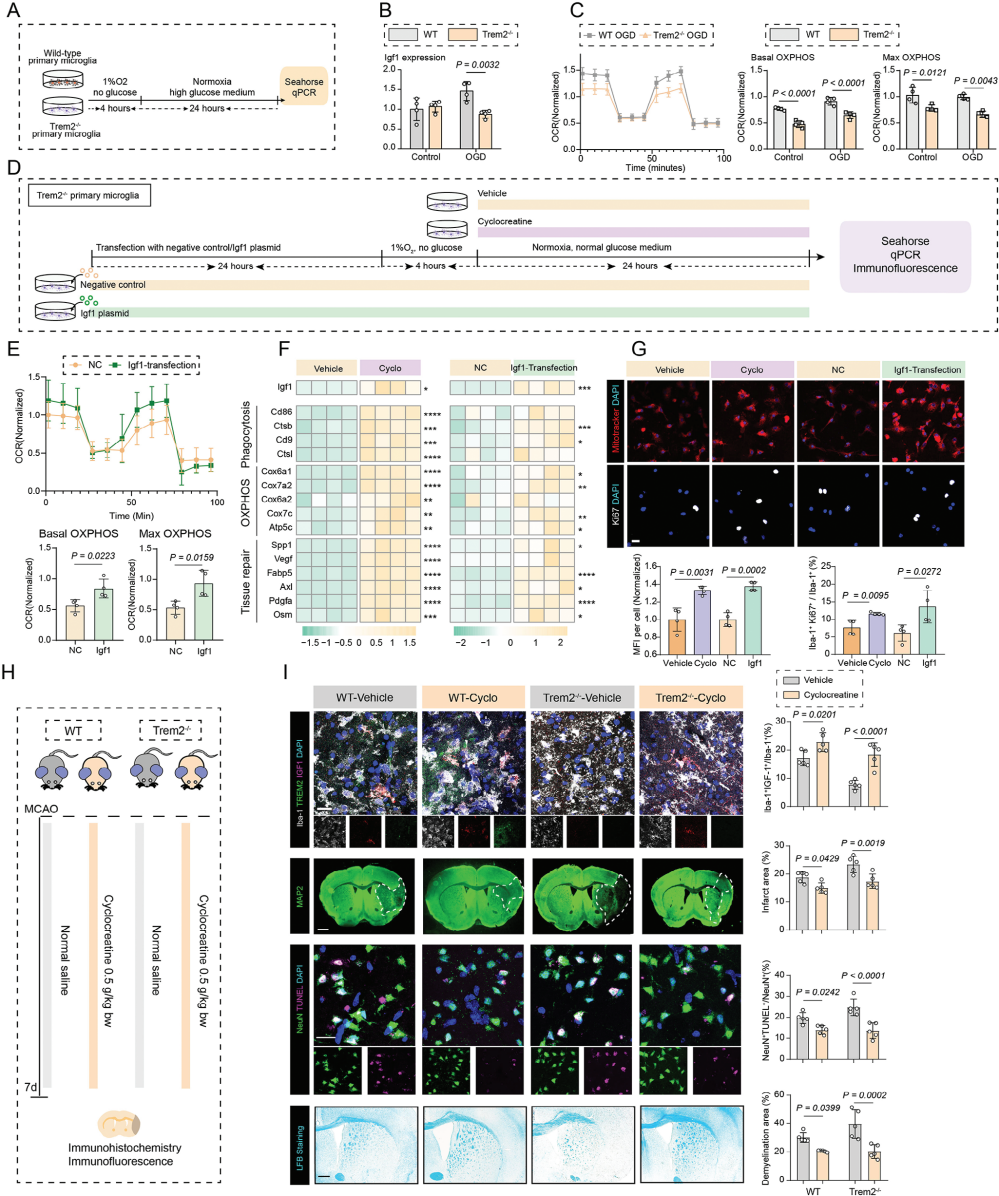
Overexpression of Igf1 or supplementation of cyclocreatine boosts microglial OXPHOS and shows neuroprotective effects. A) Schematic plot of the workflow in wild‐type Control, Trem2^−/−^ Control, wild‐type OGD/R, Trem2^−/−^ OGD/R in vitro. B) Expression level of Igf1 in wild‐type Control, Trem2^−/−^ Control, wild‐type OGD/R, Trem2^−/−^ OGD/R detected by RT‐PCR. N = 4 per group, mean ± SD, two‐way ANOVA followed by Bonferroni's post hoc test. C) Oxygen consumption rate (OCR) was measured over time using a Seahorse XFe24 analyzer. Quantification of basal OCR and maximal OCR. N = 4 per group, mean ± SD, two‐way ANOVA followed by Bonferroni's post hoc test. D) Schematic plot of the workflow of Igf1 overexpression and cyclocreatine treatment in Trem2^−/−^ microglia in vitro. E) Oxygen consumption rate (OCR) was measured over time using a Seahorse XFe24 analyzer. Quantification of basal OCR and maximal OCR. N = 4 per group, mean ± SD, Igf1‐transfection versus negative control (NC). Unpaired t test. F) Heatmap showing expression levels of genes related with phagocytosis, OXPHOS and tissue repair detected by RT‐PCR. N = 4 per group, mean ± SD, Cyclo versus Vehicle and Igf‐1 transfection versus NC. * P < 0.05, ** P < 0.01, *** P < 0.001, **** P < 0.0001, unpaired t test. G) Immunofluorescence staining of Mitotracker and Ki67^+^ proliferative microglia in the four groups: Vehicle, Cyclocreatine, Negative control, Igf1 transfection. Scale Bar: 20 µm. Quantitative analysis of Mean Fluorescence Intensity (MFI) of mitotracker per cell and Ki67^+^ proliferative microglia proportion. N = 4 per group. Data are expressed as mean ± SD, unpaired t test. H) Schematic plot of the workflow of cyclocreatine treatment in wild‐type and Trem2^−/−^ mice. I. Immunofluorescence staining of TREM2^+^ IGF1^+^ Iba‐1^+^ microglia, MAP2, TUNEL^+^ NeuN^+^ apoptotic neurons, and LFB staining in mice of wild‐type Vehicle, wild‐type Cyclo, Trem2^−/−^ Vehicle, and Trem2^−/−^ Cyclo. Scale Bar: 1 mm for MAP2 staining and LFB staining and 20 µm for the rest. Quantitative analysis of IGF1^+^ Iba‐1^+^ microglia proportion, infarct area percentage, TUNEL^+^ NeuN^+^ apoptotic neurons proportion, and demyelination area proportion. N = 5 per group, two‐way ANOVA followed by Bonferroni's post hoc test.

Based on our results of microglial Igf1 expression in a Trem2‐dependent manner, we then investigated that whether overexpression of Igf1 or boosting OXPHOS might modulate microglial functions in the absence of Trem2 in vitro. To this end, two strategies were adopted: 1) Igf1 overexpression via plasmid transfection in Trem2^–/–^ cells, and 2) administration of cyclocreatine in vitro to increase cellular ATP levels in Trem2 cells.^[^ ^14^ ^]^ Interestingly, treatment of OGD/R increased microglial basal OXPHOS levels, while Trem2 deficient microglia exposed to OGD/R presented decreased Igf1 expression and dampened basal and maximal cellular OXPHOS levels in (**Figure** **6**A–C), however, the phenomena were reversed by Igf1 overexpression (Figure 6D,E). Mechanistically, overexpression of Igf1 significantly upregulated the levels of genes related with phagocytosis (Ctsl, Ctsd, Cd9, and Ctsb), OXPHOS (Cox6a2, Cox7a2, Cox6a2, Cox7c, and Atp5c), and tissue repair (Spp1, Vegf, Axl and et al.) (Figure 6F). Meanwhile, the average Mitotracker fluorescence intensity and proportion of Ki67^+^ proliferative microglia were also promoted by Igf1 overexpression (Figure 6G).

**Figure 7:**
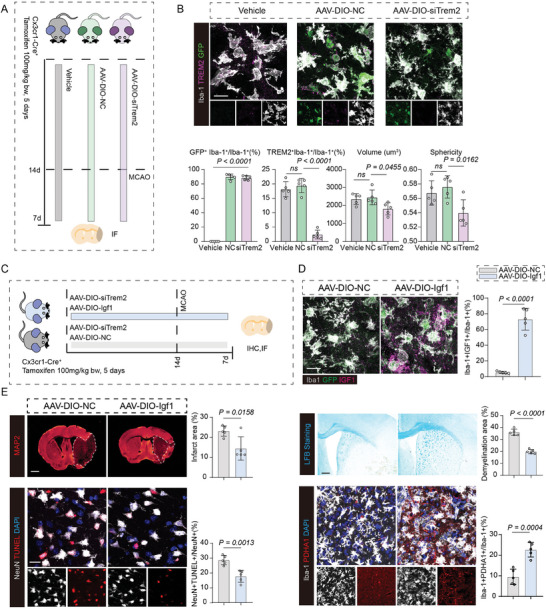
Over‐expression of microglial Igf1 protects against ischemic stroke injuries. A) Workflow of the AAV‐DIO‐siTrem2 infection strategy. B) Validation of AAV infection efficiency and Trem2 silencing efficiency. Proportions of GFP positive microglia and TREM2 positive microglia were calculated, and morphological features of microglia across different groups were analyzed. N = 5, mean ± SD, one‐way ANOVA followed by Bonferroni's post hoc test. Scale Bar: 20 um. C) Workflow of the AAV‐DIO‐Igf1 overexpression strategy under Trem2 silencing. D) AAV‐DIO‐Igf1 infection boosts microglial Igf1 expression in ischemic stroke. Proportions of IGF1 positive microglia were analyzed between AAV‐DIO‐NC and AAV‐DIO‐Igf1 overexpression groups. N = 5, mean ± SD, one‐way ANOVA followed by Bonferroni's post hoc test. Scale Bar: 20 um. E) Immunofluorescence staining of MAP2, NeuN^+^TUNEL^+^ apoptotic neurons, LFB staining and Iba‐1^+^PDHA1^+^ in Cx3cr1‐Cre^+^ mice injected with AAV‐DIO‐siTrem2+AAV‐DIO‐Igf1 or AAV‐DIO‐siTrem2+AAV‐DIO‐NC. Scale Bar: 1 mm for MAP2 staining and LFB staining and 20 µm for NeuN/TUNEL staining. Quantitative analysis of infarct area percentage, TUNEL^+^ NeuN^+^ apoptotic neurons proportion, and demyelination area proportion. N = 5 per group, mean ± SD, unpaired t test.

Similar with the results from Igf1 overexpression, cyclocreatine supplementation enhanced microglial OXPHOS, phagocytosis, proliferation and neuroprotective properties (Figure 6F,G), and also increased the expression of Igf1 in microglia (Figure 6F). In line with the findings in vitro, cyclocreatine administration could decrease the number of apoptotic neurons, and alleviate cerebral infarction and demyelination, as well as promoting the proportion of IGF1^+^ microglia, even in Trem2^–/–^ mice (Figure 6H,I). In order to better reveal the neuroprotective effects of boosting microglial Igf1 expression on stroke outcomes, AAV‐DIO‐siTrem2 combined with AAV‐DIO‐Igf1 were simultaneously injected into Cx3cr1‐Cre^+^ mice brains prior to MCAO. Immunofluorescence data revealed that a high efficiency of virus infection in microglia (≈90% characterized by GFP‐positive microglia). Microglia with Trem2 silencing exhibited smaller cell size and reduced cellular sphericity, consistent with our previous results. Under circumstances of Trem2 silencing, AAV‐DIO‐Igf1 significantly boosted the expression of Igf1 in microglia (**Figure** **7**A–D). Also, it can be revealed that the infarction and demyelination areas have been reduced after microglial Igf1 over‐expression, along with lower percentage of apoptotic neurons (TUNEL^+^NeuN^+^ cells) and enhanced expression of PDHA1 in microglia. These results further demonstrate that specifically boosting microglial Igf1 expression could potentially alleviate ischemic stroke injuries, even in the absence of Trem2 (Figure 7E).

## 3 Discussion

In the present study, we first identified a microglial subcluster up‐regulated after ischemic stroke, characterized by enhanced OXPHOS and a molecular signature of upregulated Trem2, Igf1, as well as other phagocytosis‐associated molecules. Interestingly, repopulated microglia after CSF1R inhibition resembled a similar transcriptional profile following ischemic stroke and could largely attenuate the detrimental effects of microglial depletion after MCAO. Igf1 serves as one of the most important downstream molecules of Trem2. Furthermore, overexpression of Igf1 or supplementation of cyclocreatine exhibited neuroprotective properties, under circumstances of Trem2 deficiency, leading to a reversal of ischemic brain damage induced by Trem2 knockout. Our findings indicated the importance of Trem2‐Igf1 axis in regulation of microglial activities, particularly those related with microglial glucose metabolism, and further shed light on the therapeutic potentials of targeting microglial immunometabolism in ischemic stroke.

Emerging evidence has revealed the importance of disease‐associated microglia (DAM) in Alzheimer's disease and aging,^[^ ^15^ ^]^ however, little was known in stroke. In this study, using snRNA‐seq, we identified the microglial subcluster in cerebral ischemia, characterized by significant up‐regulation of Trem2 and Igf1, enhancement of OXPHOS and microglial activation, along with characteristics such as phagocytosis and proliferation, consistent with previous studies.^[^ ^12^ ^]^ Interestingly, this microglial subcluster exhibited neuroprotective effects on surrounding cells via secretion of various ligands including IGF1, implying that activation of this microglial subcluster could be an endogenous protective mechanism toward ischemic injury. We then demonstrated that microglial depletion resulted in larger infarct lesions and worse neurological function, suggesting an indispensable role of microglia in recovery following ischemic stroke.^[^ ^3^, ^16^ ^]^ Notably, repopulated microglia after depletion also exhibited a transcriptional signature with up‐regulated Igf1 and Trem2 following ischemic circumstances, and subsequently exerted neuroprotective effects. Using the signature of differentially expressed genes in microglia, we also confirmed similar transcriptional patterns and the neuroprotective crosstalk in previous scRNA‐seq datasets,^[^ ^17^ ^]^ providing evidence of the rigor of our findings. Thus, the protective effects of this Igf1 and Trem2 high‐expressing microglial subcluster potentially involves reprogramming of microglial metabolism and functions, in which hub molecules, such as Trem2, Igf1 and others, play crucial roles.

Several studies have shown that depletion of Trem2 could exacerbate ischemic brain damage, leading to impaired neurological recovery and larger ischemic lesion volumes, possibly through the regulation of microglial phagocytotic abilities and neuroinflammatory responses. Correspondingly, the neuroprotective role Trem2 plays may be associated with alleviating neuroinflammation and neuronal apoptosis processes.^[^ ^18^ ^]^ Additionally, Trem2 has been implicated in phagocytosis processes and glycolipid metabolism in microglia.^[^ ^19^ ^]^ Moreover, a study demonstrated that microglial TREM2, along with its ligand, forms a ligand‐receptor complex that facilitates the phagocytosis of damaged cells, contributing to tissue repair.^[^ ^20^ ^]^ Correspondingly, increased levels of TREM2 in microglia protected dopaminergic neurons from apoptosis, resulting in improved motor ability in mice with Parkinson disease.^[^ ^21^ ^]^ In line with previous studies, we demonstrated that Trem2 deficiency contributed to ischemic brain injuries, accompanied by the downregulation of microglial OXPHOS and dysfunction of proliferation and phagocytosis. Based on these earlier findings and our results, we proposed that Trem2^+^ microglia exert their neuroprotective roles via enhancing microglial phagocytosis of myelin debris and apoptotic neurons, ameliorating neuroinflammation processes, and releasing neurotrophic factors. We next sought to further elucidate the mechanisms that Trem2 regulates microglia‐associated immunometabolism in ischemic stroke models. We first identified that upregulation of Igf1 was highly dependent on Trem2 both in vivo and in vitro, suggesting that Igf1 may serve as a potential downstream signal of Trem2 in ischemic stroke. The signaling pathways of TREM2‐DAP10/DAP12 could contribute to recruitment of tyrosine‐protein kinase SYK and phosphatidylinositol 3‐kinase (PI3K), triggering a series of protein and lipid phosphorylation cascades which contribute to alterations in cellular activities.^[^ ^22^ ^]^ Additionally, various neuropeptides have been suggested to regulate the expression and production of IGF1.^[^ ^23^ ^]^ Studies have also demonstrated that CD11c^+^ microglia highly expressed IGF1 to alleviate neurological pathologies.^[^ ^24^ ^]^ Given the pivotal role of TREM2 in regulating cellular signaling pathways, such as CD11c in microglia^[^ ^25^ ^]^ and peptide formation and kinase cascades, we hypothesize that TREM2 may activate other signaling pathways, such as CD11c‐related pathways and various kinase cascades, to induce the expression of Igf1 mRNA and subsequently enhance IGF1 production. However, further investigation is needed to elucidate these potential mechanisms. IGF1 is a 70‐amino acid polypeptide hormone,^[^ ^26^ ^]^ which is reported to be involved in development, proliferation, pro‐survival, anti‐aging, and exerts neuroprotective effects in a variety of diseases.^[^ ^24^, ^27^ ^]^ Meanwhile, IGF1 has been reported to promote mitochondrial function, including OXPHOS coupling efficiency and ATP production,^[^ ^28^ ^]^ improve metabolism and eventually exert mitochondrial protection effects.^[^ ^28^, ^29^ ^]^ As expected, our data further indicated that overexpression of Igf1 in Trem2 deficient microglia could enhance the levels of OXPHOS, promote microglial phagocytosis and proliferation, and subsequently reverse the effects depletion of Trem2 exerts on microglia. From our perspectives, boosting Igf1 expression could on the one hand, enhance microglial activities. On the other hand, enhanced secretion of IGF1 could promote survival of neurons based on its neurotrophic properties. These data raise the novel concept of Trem2‐Igf1 signaling axis in orchestrating microglial OXPHOS metabolism profiles and functions in ischemic stroke.

Cyclocreatine, a creatine analog that easily cross blood‐brain barriers and enter the brain, can maintain cellular ATP levels in need of energy,^[^ ^14^ ^]^ and has shown protective effects in a variety of diseases, such as heart failure,^[^ ^30^ ^]^ prostate cancer,^[^ ^31^ ^]^ and neurodegenerative disorders.^[^ ^32^ ^]^ Our study further confirmed that, in either WT or Trem2 deficient microglia, supplementation of cyclocreatine under the circumstance of hypoxia in vitro was beneficial for maintaining normal functions and metabolic profiles via the upregulation of Igf1 expression. Remarkably, administration of cyclocreatine after MCAO facilitated microglia shifting toward Igf1 high‐expression phenotype and further attenuated neurologic deficits, even in the absence of Trem2. Notably, besides pharmacologically improving microglial metabolic profiles, genetically enhancing Igf1 expression specifically in microglia also protects from ischemic stroke injuries. Altogether, our study indicates that supplementation of cyclocreatine targeting microglial metabolism may be promising for therapeutic intervention in ischemic stroke, even in the absence of Trem2, the mechanisms of which is closely associated with regulation of Igf1 expression.

In summary, we identified a microglial subcluster, which was specifically upregulated in ischemic lesions of MCAO models, with differentially altered glucose and ATP metabolism and neuroprotective effects on surrounding cells. This study, in addition, strongly suggested that Trem2‐Igf1 signaling axis orchestrated this microglial phenotype shift during ischemic stroke, and immunometabolism underlying microglial transformation could be pharmacologically or genetically modulated. We further demonstrated the therapeutic potentials of cyclocreatine in ischemic stroke, by enhancing microglial OXPHOS and facilitating its conversion from dystrophic microglia toward neuroprotective state, possibly via activation of Igf1. Thus, understanding how to activate Trem2‐Igf1 signaling axis in microglia plays crucial roles, is key to harness neuroprotective potential for the treatment of ischemic stroke.

### 3.1 Limitations of the Current Study

The study has some limitations. First, to add novelty of the manuscript, we've focused more on Trem2‐Igf1 signaling axis and microglia repopulation strategy, along with polishing the writing of the manuscript. We acknowledge that microglia responses to ischemic stroke vary significantly on different days after injury, and cannot be entirely captured at a single time point (we mainly focused on day 7 in this study). To compensate for this, we have included more data of both immunofluorescence and single cell RNA‐Sequencing at different time points after stroke. Additionally, it still remains unclear how Trem2 regulates Igf1 expression. Thus, the detailed mechanisms that Trem2 exerts impacts on Igf1 expression need to be further elucidated. Furthermore, molecular quantification methods such as RNA‐scope and western blotting could be applied to address these gaps, which we aim to improve upon in future studies.

## 4 Experimental Section

### Animals

C57BL/6 male mice (wild‐type; 20–25 g; 8–10 weeks) were obtained from Hunan SJA Laboratory Animal Co. Ltd, Hunan, China. Trem2 mice were kindly gifted by prof. Marco Colonna at Washington University, St. Louis. Cx3cr1^CreER^ mice (B6.129P2(Cg)‐Cx3cr1^2.1(cre/ERT2)Litt/WganJ^) were obtained from The Jackson Laboratory (Bar Harbor, ME, USA).

All animal surgeries were approved by the Animal Care Committee of Tongji Medical College, Huazhong University of Science and Technology. All mice were randomly divided into different treatment groups. Researchers were blinded in the morphological analysis.

### Primary Microglia Culture

Primary mouse microglia were extracted from brains of 0–3 days postnatal mouse pups and cultured as previously described.^[^ ^33^ ^]^ Briefly, mixed glia cultures were obtained from brains of P1‐2 neonatal mice. The brains of the neonatal mice were cut up and digested with 0.125% trypsin. The isolated cells were cultured in poly‐d‐lysine coated flask with DMEM/F12 containing 10% heat‐inactivated fetal bovine serum. Then the cells were cultured in a humidified incubator at 37 °C with 5% CO_2_ for 10–12 days.

### Microglia Depletion and Repopulation Strategy

Microglia depletion and repopulation are conducted with PLX3397 (Selleck, S7818) as previously described.^[^ ^34^ ^]^ Briefly, mice were subjected to chows containing 290 mg k^−1^g for 21 consecutive days before surgery. Then after surgery, mice for complete microglia depletion continued to be fed with those PLX3397 chows, while those for microglia repopulation were returned to normal chow. 1 day, 3 days and 7days after ischemic stroke, brains of mice were harvested for subsequent analysis.

### Middle Cerebral Artery Occlusion

Middle cerebral artery occlusion (MCAO) model in mice was established as previously described with little modifications.^[^ ^35^ ^]^ Briefly, mice were anesthetized with isoflurane and a monofilament with diameter of ≈0.18 mm (for mice weighted 20–25 g) was used to block the cerebral blood flow in right hemisphere for 60 min. The sham‐operated group received the same surgery without monofilament to block blood flow in middle cerebral artery. After surgery, mice were fed in SPF environment and supplied with sufficient food and water.

### Behavioral Tests

Neurological deficits in mice were evaluated with modified neurological severity score (mNSS) as previously described.^[^ ^36^ ^]^ Briefly, this mNSS system is composed of motor abilities, reflex test and balance tests and the total score of neurological deficits ranges from 0 (completely no symptoms) to 14 (most severe). mNSS tests were conducted before surgery, 1d, 3d and 7d after surgery.

### Cyclocreatine Administration

Cyclocreatine was orally administered to both wild‐type and Trem2 mice with a gastric needle at the dosage of 0.5 g kg^−1^ for 7 consecutive days after MCAO surgery. The dose and administration strategy were implemented as previously described.^[^ ^37^ ^]^ Mice from vehicle group received the same of amount of normal saline, also for 7 consecutive days after MCAO surgery.

### Tamoxifen Injection

2‐month old male Cx3cr1‐Cre^+^ transgenic mice were intraperitoneally injected with tamoxifen (MCE) once a day (100 mg k^−1^g body weight, dissolved in corn oil at a final concentration of 20 mg ml^−1^) for 5 consecutive days according to previous studies.^[^ ^38^ ^]^

### Adeno‐Associated Virus Stereotactic Injection

Stereotactic injection of adeno‐associated virus was performed according to previous studies with some modifications.^[^ ^38^, ^39^ ^]^ Briefly, mice were anesthetized with isoflurane and fixed in stereotactic frame. The virus was stereotaxically injected into the right brain cortex (AP 0.02 mm, ML −3 mm, DV −2 mm). Total volume of the virus per mouse injected was 0.6–0.8 ul at a rate of 0.1 ul min^−1^. Keep the syringe still until 10 min after the end of the infusion to let the virus perfuse in the brains. Mice from vehicle group and NC group received the same of amount of normal saline and negative control vector, respectively. After injection, keep the mice in SPF conditions with sufficient food and water and suitable temperature. Two weeks after the virus injection, these mice received subsequent surgeries.

### Single‐Nuclei RNA‐Sequencing

Brain samples of hemisphere from sham‐operated mice and of ischemic hemisphere from MCAO mice were collected for snRNA‐seq analysis. Totally 61 426 cells were included. CellRanger Single Cell Software (v 3.1.0)^[^ ^40^ ^]^ was utilized to process single‐cell FASTQ raw sequencing read of each sample. Then the individual datasets from each sample were aggregated via the “aggr” command with no subsampling normalization.

Seurat package (v 4.04)^[^ ^41^ ^]^ was used for subsequent analysis. Cells which express fewer than 200 genes were excluded, and cells with > 90% of the maximum genes were also discarded to avoid “doublet” events. In order to prevent mitochondrial contamination, we identified cells with > 7.5% mitochondrial genes as poor‐quality cells and excluded. The expression matrix was normalized using the normalize Data function and the top 2000 variable genes were calculated with FindVariableFeatures. The Seurat object was then scaled with the scaleData function and principal component analysis (PCA) was further implemented. The top 20 principal components were implemented for the unsupervised clustering. “FindIntegrationAnchors” was utilized to remove batch effects among different samples. FindNeighbors function was used to construct the k‐nearest neighbors' graph, and FindClusters function was used to iteratively group nuclei (resolution = 0.5). The cell type of each cluster was identified based on the cellular markers identified based on FindAllMarkers function. For the re‐clustering of microglia/macrophages, top 10 principal components were used for the clustering. R package monocle (2.20.0),^[^ ^42^ ^]^ clusterProfiler (v 4.0.5),^[^ ^43^ ^]^ msigdbr (7.4.1), ggplot2 (v 3.3.5) and Pheatmap (1.0.12) were utilized for subsequent analysis.

### Fluorescence‐Activated Cell Sorting

Fluorescence‐activated cell sorting (FACS) was performed on Moflo XDP flowcytometry cell sorting (Beckman Coultre). Briefly, ischemic brain hemispheres of MCAO mice and the hemispheres of sham‐operated mice were harvested, placed in ice‐cold tissue storage buffer (Miltenyi) and enzymatized with Enzyme P and Enzyme A for 30 min. Then myelin debris were removed, and cells pellets were re‐suspended with FACS buffer, blocked with Fc block for 15 min and stained with CD45 and CD11b antibodies for 30 min on ice and protected from light. Viable 7‐AAD^−^CD11b^+^CD45^int^ microglia cells were used for RNA‐Sequencing analysis.

### Bulk RNA‐Sequencing

Briefly, total RNA was extracted from FACS sorted microglia by RNeasy Micro Kit (QIAGEN, Germany) according to manufacturer's protocol. Then total RNA was qualified and quantified by NanoDrop and Agilent 2100 bioanalyzer. RNA of high‐quality was amplified with oligo‐dTs and dNTPs, then further transcribed into cDNA. The final cDNA library was amplified into DNA nanoball (DNB) and loaded into the pattern nanoarray. Finally, single‐end 50 bases read were generated on BGISEQ500 platform (BGI‐SHENZHEN).

### Ingenuity Pathway Analysis

Ingenuity pathway analysis (IPA) were implemented according to the manufacturer's instructions. In this paper we had mainly focused on Canonical Pathways in its core analysis functions, and pathways with P value < 0.05 were considered significant.

### Immunofluorescence Staining

Briefly, mice brain sections were incubated with primary antibodies overnight at 4 °C, washed for 5 min for 3 times, and then incubated with secondary antibodies at room temperature for 1 h in the dark. Images were obtained with a confocal microscope (OLYMPUS, FV1200). The border of infarction was determined by the morphology of NeuN^+^ neuron and Iba‐1^+^ microglia, and the peri‐infarct area was defined as the tissue that covers a radial distance of 200–300 mm from the border of the infarct as previously reported.^[^ ^3^ ^]^ For each mouse, 4–5 microscopic fields were utilized for statistics analysis.

### Magnetic Activated Cell Sorting

Microglia were sorted using CD11b microbeads following manufacturer's instructions. Briefly, CD11b microbeads‐conjugated antibodies were added to the cell suspension at a dilution of 1:10 and incubated on ice for 10–15 min. Then the cell suspension containing antibodies were transferred onto an MS magnetic column and injected into a 15 ml centrifuge tube. Supernatant was carefully removed after centrifugation of 300 g for 5 min at 4 °C. After that, 350 ul RLT buffer was added to the cell pellet and stored at −80 °C for PCR analysis.

### Oxygen‐Glucose Deprivation‐Reperfusion

Oxygen‐glucose deprivation‐reperfusion (OGD/R) model in primary mouse microglia in vitro was conducted as previously described.^[^ ^44^ ^]^ Briefly, OGD was performed with medium replaced with no‐serum contained PBS and incubated in a non‐CO_2_ incubator for 4 h at 1% O_2_ concentration. Reperfusion was performed with DMEM/High glucose medium containing 20% FBS and incubation in a normal incubator containing 20% O_2_ and 5% CO_2_ for 24 h.

### Seahorse Metabolic Analysis

Briefly, primary mouse microglia were seeded into XFe24 cell culture plates at a density of 80 000 cells per well and Mitostress kit (Agilent) was used to measure oxygen consumption rates. Cell culture medium was replaced with fresh running buffer (XF DMEM medium plus 10 mM glucose, 2 mM glutamine and 1 mM pyruvate) and incubated in a non‐CO_2_ incubator for 60 min. 1.5 µM oligomycin, 2 µM FCCP and 0.5 µM rotenone plus antimycin A were added to the sensor cartridges in calibration processes. Values of OCR are normalized to the cell number in each well. The values of the first measurement points of OCR for WT‐control or negative control‐transfected microglia were normalized to 1.

### RNA Extraction and Real‐Time PCR

Total RNA from primary microglia or MACS‐sorted microglia were extracted by TRIzol or by RNeasy Micro Kit (QIAGEN, Germany) following manufacturer's instructions. Then cDNA was reversely transcribed from 1ug of RNA using PrimeScript RT Master Mix (TAKARA, RR036A). A total of 20 µL reaction system was prepared for quantitative RT‐PCR using Hieff qPCR SYBR Green Master Mix (YEASEN, 11201ES03).

Real‐time PCR system (CFX96, Bio‐Rad) was used for all reactions based on manufacturer's protocol. The expression levels of target genes were normalized to β‐actin and calculated using 2ΔΔ^Ct^ method.

### Plasmid Transfection

Plasmid of mouse Igf1 and negative control were purchased from Origene. The whole process of transfection was conducted according to manufacturer's protocol, with reference to previous studies of plasmid transfection in primary mouse microglia with some modifications.^[^ ^45^ ^]^ Briefly, cells were seeded in cell culture plate for 24 h to be adhered to the plate bottom. For each well (24‐well plate), 0.5 ug of plasmid DNA was diluted into 50ul serum‐free Opti‐MEM. Meanwhile, dilute 2.5 ul of SuLiTran transfection into 50 ul of serum‐free Opti‐MEM medium. Add the diluted DNA into the diluted SuLiTran reagent with ratio of 1:1 and mix well. Add this 100 ul mixture prepared onto seeded cells. Incubate cells at 37 °C. 6–8 h after, replace the current cell culture medium with fresh pre‐warmed medium containing FBS. 24 h after transfection, cells were utilized for further treatment and analysis.

### Luxol Fast Blue (LFB) Staining

Luxol fast blue (LFB) staining was utilized to visualize myelination and determine myelin loss in MCAO models. Briefly, 20 um brain slices were stained with pre‐warmed 0.1% LFB dye (G1030, Servicebio) at 60 °C for 6–8 h. Then the slices were differentiated alternately with 0.05% lithium carbonate and 70% ethanol, followed by dehydration subsequently with 75%, 90% and 100% ethanol. Finally, these slices were sealed with neutral resin and were captured by a microgscope (BX51, Olympus, Japan). The percentage of demyelination was calculated as the demyelinating area proportion as previously described.^[^ ^46^ ^]^

### Mitotracker Staining

After OGD/R, primary microglial cells were stained with 200 nM mitotracker (YEASEN) at normal cell culture conditions for ≈30 min. After mitotracker staining, cells were immediately harvested on coverslip and images of fluorescence were captured with a confocal microscope (OLYMPUS, FV1200, Japan).

### Statistics Analysis

Data in the figures are presented in the form of mean ± SD, and normalization and transformation methods are shown in respective figure legends. The number of animals and in vitro replicates for each experiment in different groups are also shown in the figure legends. Unpaired two‐sided Student's t‐test, one‐way ANOVA or two‐way ANOVA followed by Bonferroni test were performed by GraphPad Prism software version 9. Exact P values of statistics analysis were directly shown on the Figures, and P value < 0.05 was considered statistically significant.

## Conflict of Interest

The authors declare no conflict of interest.

## Author Contributions

S.Y., C.Q., M.C., D.S.T., and W.W. performed conceptualization; S.Y., M.C., M.H.D., L.Q.Z., Y.H.C., Y.T., H.Z., X.W.P., and L.C., performed methodology; S.Y., C.Q., M.C., M.H.D., L.Q.Z., Y.H.C., Y.T., H.Z., X.W.P., and L.C., performed investigation; L.J.W., D.S.T., and W.W., performed visualization; C.Q., D.S.T., and W.W., performed funding acquisition; C.Q., S.Y., M.C., L.J.W., D.S.T., and W.W., performed project administration; L.J.W., D.S.T., and W.W., performed supervision; C.Q., S.Y., M.C., and M.H.D. wrote the original draft; L.J.W., and D.S.T., wrote, reviewed and edited.

## Supporting information

Supporting Information

Supporting Information

Supporting Information

Supporting Information

Supporting Information

Supporting Information

Supporting Information

## Data Availability

The data that support the findings of this study are available on request from the corresponding author. The data are not publicly available due to privacy or ethical restrictions.
