# Supplementary material for: TREM2‐IGF1 Mediated Glucometabolic Enhancement Underlies Microglial Neuroprotective Properties During Ischemic Stroke
Source: Adv Sci (Weinh). 2023 Dec 27;11(10):2305614. doi: 10.1002/advs.202305614 (PMC10933614; doi:10.1002/advs.202305614)
Supplement: Supplementary file 1 — Supporting Information [file ADVS-11-2305614-s007.pdf]

ADVANCED SCIENCE
Open Access

# Supporting Information

for *Adv. Sci.*, DOI 10.1002/advs.202305614

TREM2-IGF1 Mediated Glucometabolic Enhancement Underlies Microglial Neuroprotective Properties During Ischemic Stroke

*Sheng Yang, Chuan Qin, Man Chen, Yun-Hui Chu, Yue Tang, Luo-Qi Zhou, Hang Zhang, Ming-Hao Dong, Xiao-Wei Pang, Lian Chen, Long-Jun Wu, Dai-Shi Tian* and Wei Wang**

| Reagents | Source | Identifier |
|---|---|---|
| Antibodies | | |
| CD11b-FITC | Biolegend | Cat#101206 |
| CD45-APC | Biolegend | Cat#147708 |
| Iba-1 | WAKO | Cat #019-19741 |
| Iba-1 | Abcam | Cat #NB-100-1028 |
| APOE | Abcam | Cat #ab183597 |
| IGF1 | Abcam | Cat #ab9572 |
| TREM2 | R&D | Cat #MAB17291-SP |
| LGALS3 | Biolegend | Cat #125401 |
| CLEC7A | Invivogen | Cat#mabg-mdect |
| MAP2 | Proteintech | Cat# 17490-1-AP |
| CD11b microbeads | Miltenyi biotec | Cat#130-093-636 |
| OLIG2 | R&D | Cat# AF2418 |
| Citrate Synthase (CS) | Abcam | Cat #ab129095 |
| PDHA1 | Abcam | Cat #ab168379 |
| NeuN | CST | Cat #36662 |
| Ki67 | CST | Cat #9129 |
| CD68 | Bio-rad | Cat#MCA1957 |
| Chemicals, peptides, and recombinant proteins | | |
| Seahorse glycolytic rate assay | Agilent | Cat #103344-100 |
| Seahorse mitostress assay | Agilent | Cat #103015-100 |
| XFe testing plate | Agilent | Cat #102342-100 |
| Seahorse XF DMEM testing medium | Agilent | Cat #103575-100 |
| Seahorse XF Glucose solution | Agilent | Cat #103577-100 |
| Seahorse XF Pyruvate solution | Agilent | Cat #103578-100 |
| Seahorse XF glutamine solution | Agilent | Cat #103579-100 |
| Cyclocreatine | MCE | Cat#HY-W017540 |
| Mitotracker | YEASEN | Cat #40740ES50 |
| Adult brain dissociation kit | Miltenyi Biotec | Cat #130-107-677 |
| Rneasy micro kit | Qiagen | Cat #74004 |
| 7-AAD | BD Bioscience | Cat #559925 |
| MS column | Miltenyi Biotec | Cat #130-042-201 |
| TUNEL (TMR Red) | Roche | Cat #12156792910 |
| TUNEL (Fluoresence) | Roche | Cat #11684795910 |
| DAPI | Beyotime | Cat #P0131 |
| TRIzol | ThermoFisher | Cat#15596026 |
| LFB staining kit | ServiceBio | Cat #G1030 |
| PrimeScript™ RT Master Mix | TAKARA | Cat #RR036A |
| Hieff® qPCR SYBR Green Master Mix | YEASEN | Cat # 11201ES03 |

| | | |
|---|---|---|
| PLX3397 | SelleckChem | S7818 |
| Quickblock Primary Antibody Dilution Buffer | Beyotime | Cat # P0262 |
| Quickblock Secondary Antibody Dilution Buffer | Beyotime | Cat # P0265 |
| Igf1 Mouse tagged plasmid | Origene | Cat # MG227062 |
| Adeno-associated virus | | |
| pAAV-EF1a-DIO-Igf1-P2A-EGFP-WPRE | OBiO Technology ( Shanghai ) | N/A |
| pAAV-CBG-DIO-EGFP-miR30shRNA(Trem2)-WPRE | OBiO Technology ( Shanghai ) | N/A |
| | | |
| Experimental models: Organisms/strains | | |
| WT C57BL/6 mice | Hunan SJA Laboratory Animal Co. Ltd, Hunan, China. | Hunan SJA Laboratory Animal Co. Ltd, Hunan, China. |
| Trem2$^{-/-}$ mice | This paper | Prof. Marco Colonna, Washington University |
| Cx3cr1CreER mice(B6.129P2(Cg)-Cx3cr1tm2.1(cre/ERT2)Litt/WganJ) | The Jackson Laboratory | Stock No: 021160 |
| | | |
| Software | | |
| GraphPad Prism 9 | GraphPad Software. Inc. | https://www.graphpad.com/ |
| Imaris 9 | Bitplane | https://imaris.oxinst.com/ |
| R 4.04 | R | https://www.r-project.org/ |
| ImageJ (Fiji) | NIH | https://imagej.nih.gov/ij/ |
| R package Seurat 4 | Stuart et al, 2019[1] | https://satijalab.org |
| R package ggplot2 v3.35 | Wickham, 2019 | https://cran.r-project.org |
| R package clusterProfiler v4.05 | Yu et al, 2021[2] | https://yulab-smu.top/biomedical-knowledge-mining-book/ |
| R package monocle v3 | Cole et al, 2014[3] | Monocle (cole-trapnell-lab.github.io) |
| R package CellChat | Jin et al, 2021[4] | http://www.cellchat.org/ |
| Ingenuity pathway analysis | QIAGEN | https://www.qiagen.com/ |
| | | |
| Public dataset | | |
| GSE174574 | Zheng et al, 2021[5] | https://www.ncbi.nlm.nih.gov/geo/query/acc.cgi?acc=GSE174574 |
| GSE189432 | Beuker et al, 2021[6] | https://www.ncbi.nlm.nih.gov/geo/query/acc.cgi?acc=GS |

| | | |
|---|---|---|
| | | E189432 |


1. Stuart T, Butler A, Hoffman P, et al. Comprehensive Integration of Single-Cell Data. Cell. 2019 2019/06/13/;177(7):1888-902.e21.

2. Wu T, Hu E, Xu S, et al. clusterProfiler 4.0: A universal enrichment tool for interpreting omics data. The Innovation. 2021 2021/08/28/;2(3):100141.

3. Trapnell C, Cacchiarelli D, Grimsby J, et al. The dynamics and regulators of cell fate decisions are revealed by pseudotemporal ordering of single cells. Nature Biotechnology. 2014 Apr;32(4):381-U251.

4. Jin S, Guerrero-Juarez CF, Zhang L, et al. Inference and analysis of cell-cell communication using CellChat. Nature Communications. 2021 Feb 17;12(1).

5. Zheng K, Lin L, Jiang W, et al. Single-cell RNA-seq reveals the transcriptional landscape in ischemic stroke. J Cereb Blood Flow Metab. 2022 Jan;42(1):56-73.

6. Beuker C, Schafflick D, Strecker JK, et al. Stroke induces disease-specific myeloid cells in the brain parenchyma and pia. Nat Commun. 2022 Feb 17;13(1):945.

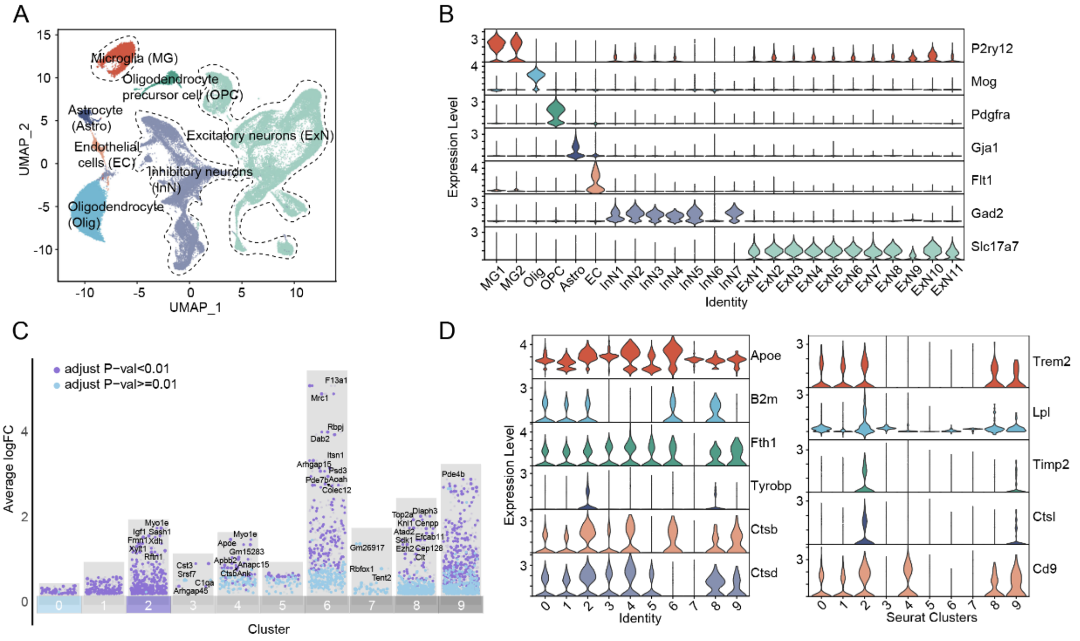


**Figure S1 Single-nuclei RNA sequencing identifies cell clusters and microglial subclusters in sham and MCAO mice brains.**

A. UMAP and unsupervised clustering of all cells isolated from brain tissue of sham mice and ischemic hemispheres MCAO mice.

B. Violin plot showing marker genes expression in different cell clusters.

C. Significantly upregulated genes in different microglial subclusters.

D. Violin plot showing marker genes expression in different microglial subclusters.

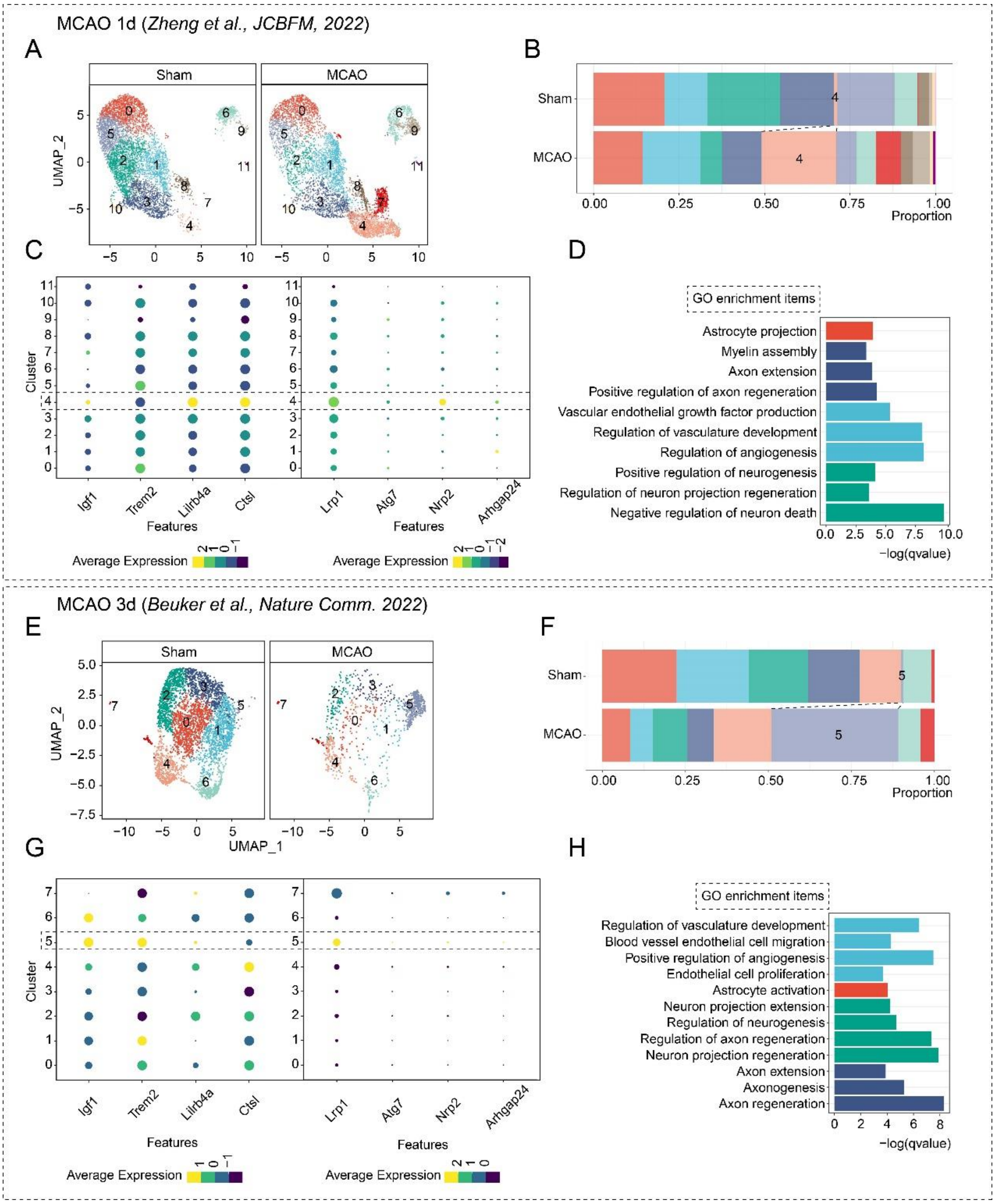


**Figure S2 Validation of microglial subcluster2 phenotype in external scRNA-seq datasets.** Re-analysis was performed on scRNA-seq datasets previously reported by Zheng et al.(upper) and Beuker et al (bottom).

A. UMAP of different cell clusters between Sham-operated group and MCAO 1d group.
B. Barplot showing proportions of different microglial subclusters between the two groups.
C. Dot plots showing marker genes expression in different cell clusters between Sham-operated group and MCAO 1d group.
D. GO biological processes with respect to indicated cell activities of the DEGs in Cluster 4 (MCAO 1 day).
E. UMAP of different cell clusters between Sham-operated group and MCAO 3d group.

F. Barplot showing proportions of different microglial subclusters between the two groups.
G. Dot plots showing marker genes expression in different cell clusters between Sham-operated group and MCAO 3d group.
H. GO biological processes with respect to indicated cell activities of the DEGs in Cluster 5 (MCAO 3 day).

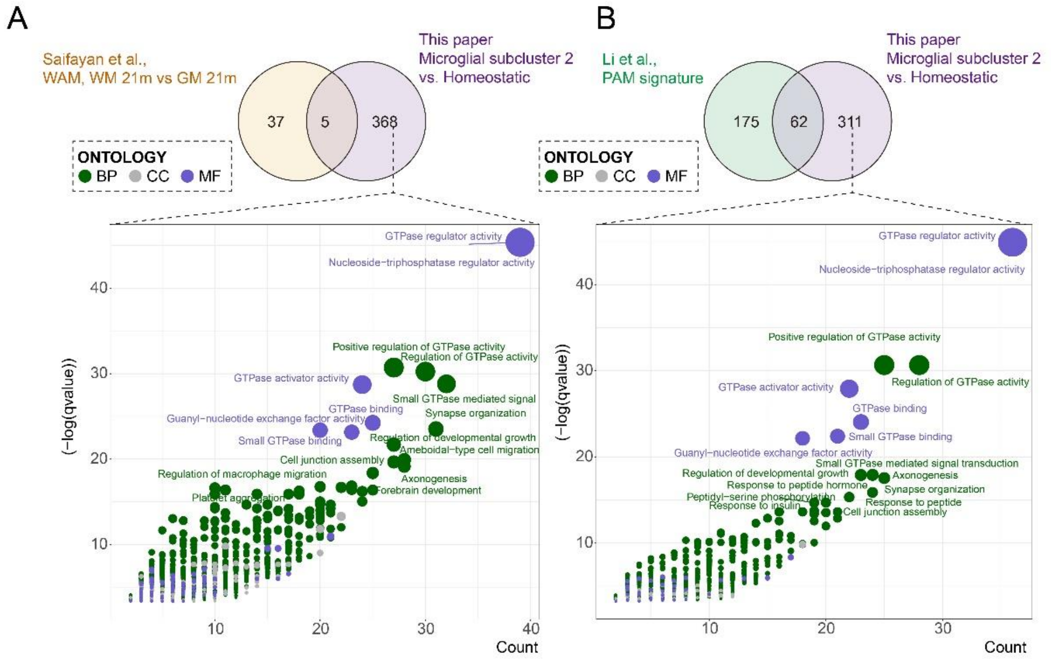


**Figure S3 Comparison of microglial subcluster 2 with WAM (A) and PAM (B).**

Number of genes that are co-expressed or exclusively expressed in microglial subcluster 2 and enriched GO items for those exclusively expressed genes were illustrated respectively.

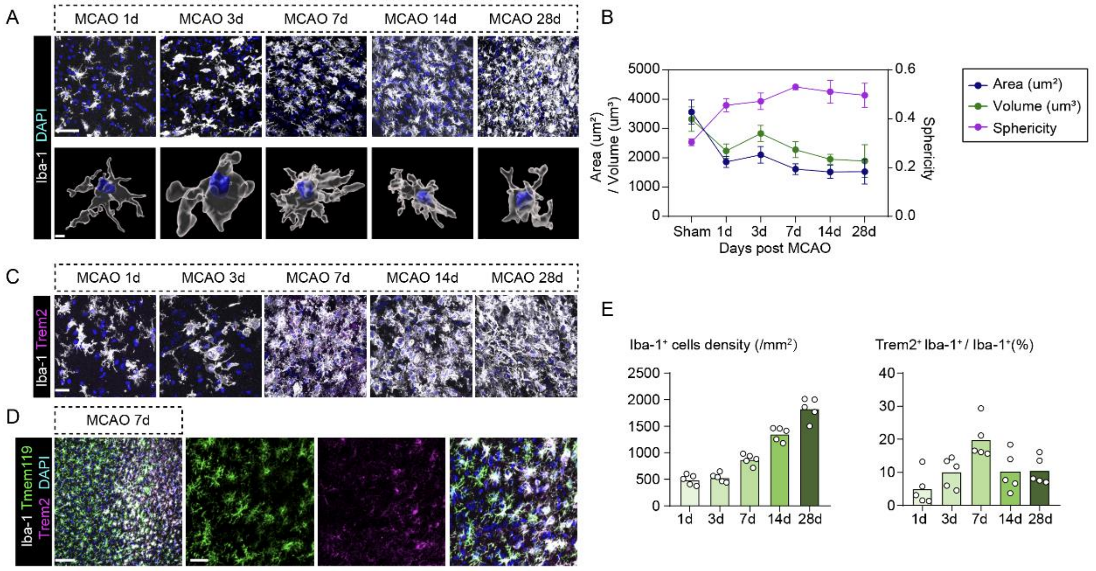


**Figure S4 Microglial morphology and density changes following MCAO.**

A. Immunofluorescent staining of Iba-1$^{+}$ microglia in mice at indicated time points after MCAO. Scale Bar: 20μm for immunofluorescent plots and 5μm for 3D reconstruction plots.

B. Morphological analysis of Iba-1$^{+}$ microglia, mean ± SD. N = 5 per group.

C. Immunofluorescent staining of Iba-1$^{+}$ Trem2$^{+}$ microglia in mice at indicated time points after MCAO. Scale Bar: 20μm. N = 5 per group.

D. Immunofluorescent staining of Iba-1$^{+}$ Tmem119$^{+}$ microglia and Iba-1$^{+}$ Trem2$^{+}$ microglia in

mice at 7 days after MCAO. Scale Bar: 100μm (left) and 20μm (right). N = 5 per group.

E. Barplots showing the Iba-1$^{+}$ microglia density at indicated time points after MCAO, and Iba-1$^{+}$ Trem2$^{+}$ cell proportion in microglia at indicated time points after MCAO. N = 5 per group.
